# Exploring perceptions of pre-clerkship students about workplace learning in the clinical learning environment at Gulf Medical University, UAE

**DOI:** 10.1186/s12909-024-05312-6

**Published:** 2024-05-13

**Authors:** Nooreen Adnan, Farah Azhar, Syeda Rubaba Azim

**Affiliations:** 1 Dow University of Health Sciences, Karachi, Pakistan; 2 Gulf Medical University , Ajman, UAE

**Keywords:** Clinical learning environment, Focus groups, Transition, Pre-clerkship, Undergraduate, Formal orientation, Experiential learning, Participation, Supervision, Learning opportunities, Feedback

## Abstract

**Background:**

Medical students view clinical workplace placements as an inspiring and motivating learning environment where active student participation is pivotal to development of students’ identity. The progress from pre-clerkship to clerkship education harbors many challenges which consist of experiential learning, adjusting to the clinical environment, and understanding roles & responsibilities. Workplace learning is underpinned by various adult learning theories including social theories,constructivism, supported participation and legitimate peripheral participation. Workplace learning course was recently initiated for pre-clerkship students at a medical university in UAE, which will enable their smooth entry into the clerkship phase of the curriculum.

**Objectives:**

The research aims to explore students’ perceptions of various domains of their clinical learning environment (CLE), highlight the challenges they face, and extract valuable feedback to improve their environment.

**Methods:**

This study was conducted qualitatively by using focus groups method in order to explore students’ perceptions of the clinical learning environment. Two focus group discussions were conducted (*n* = 8 +/-10) to determine the common challenges of workplace learning and its potential solutions. Data were analyzed using thematic analysis. The approach used to carry out this study was phenomenology, as it helps to understand the learning and behavior of these students who are undergoing this pre-clerkship training in order to transition smoothly to the clerkship phase.

**Result:**

The focus groups helped to deeply explore the perceptions of students about their clinical learning environment. It helped to reveal the challenges encountered by the students including the significance of proper orientation of staff and students, language barrier, availability of learning opportunities, and supervision quality. The focus groups provided worthwhile suggestions to improve the learning opportunities in the clinical learning environment which include orientation of the staff and students what to expect, improved supervision, mentoring and providing learning opportunities to encourage participation.

**Conclusion:**

This study attempted to identify the pre-clerkship students’ perception of their clinical learning environment and the challenges they face over there. Possible suggestions by the students included a formal orientation for the staff and students to be carried out at the beginning. Efforts should be made by clerkship directors to provide students with learning opportunities by increasing patient exposure, encouraging participation, and providing high-quality supervision.

## Introduction

Clerkship is a progression of students principally from a non-clinical environment to a clinical one towards the later years of the undergraduate medicine curriculum [1]. To enable students to have a smooth entry into the clerkship phase and help lessen the anxiety related with the transition, many medical schools have integrated workplace learning into the pre-clerkship curriculum. Hence a workplace learning course was introduced for the pre-clerkship students at Gulf medical University as well [2, 3]. Typically, the undergraduate medicine curriculum at the Gulf Medical University consists of five years with a one-year internship. The pre-clerkship phase consists of second and third year MBBS where the students will undergo clinical postings daily for four hours during their typical week at the hospital, where they are rotated in all of the major departments. After the pre-clerkship phase, the students’ progress to the clerkship phase (fourth and final year). This shift from pre-clerkship to clerkship phase raises many challenges which include employing basic clinical skills, experiential learning, adapting to the clinical environment and understanding roles and responsibilities [4]. Workplace learning course was recently introduced for the pre-clerkship students which can facilitate their smooth entry into clerkship phase of the curriculum and reduce the anxiety associated with the transition [2]. Workplace learning is a combination of individual, organizational and social processes that can together create an optimal learning environment. It is understood as applying knowledge at the workplace, already learned in the classroom known as knowledge contextualization [5].

In order for workplace learning to be practiced effectively, it is pertinent to understand its basis and relate it to the adult learning theories. Learning in a clinical workplace environment can be supported by some of the adult learning theories in literature. Though the adult learning theories suggest that self-direction is an optimal way for medical students to learn in clinical postings but in addition to self-direction, appropriate guidance from expert practitioners would greatly help the learners to accomplish what they cannot accomplish on their own, particularly those who haven’t attended clinical postings earlier [5]. Here the concept of ‘supported participation’ as proposed by Billet’s pedagogy of workplace learning is important [6]. This means participation of learners is supported by the environment in which learning occurs. The influence of the environment and the presence of experienced people at the workplace greatly modify learning at workplace and can be described as learning within the construct of social constructivism. This is where the social theories of learning can be applied [7]. The socio-cultural theorists view participatory experiences as learning by constructivism and describe different ways in which learners develop professional knowledge, skills, and more importantly, workplace identities [8]. Vygotsky aimed to match the developmental stage of the learner with the paradigm of zone of proximal development [9, 10]. This means how a learner can benefit with the support of an experienced individual at the workplace. As peripheral participants in these professional communities, students may find the amount they have to learn overwhelming, and they may struggle with their identity as a community member being in that transition stage where they are exposed first time to a clinical setting [11]. Vygotsky’s theory explains that learning is accomplished by novice learners while working with the support of experienced healthcare professionals. Additionally, Lave & Wenger hold the opinion that participation in social practice will ultimately result in learning [12, 13]. They have explained workplace learning through legitimate peripheral participation [7]. Legitimate peripheral participation can be explained as: a new learner enters the clinical workplace (community),which in this case is the second and third year pre-clerkship students, and is gradually involved in increasingly complex activities i.e., from observation to more participation and hence the novice learner becomes a part of that community, sharing many of the workplace activities. The students are mostly observing the workplace activities in the pre-clerkship phase and gradually they move towards complex activities as they progress to the clerkship phase. Wenger also proposed that social theories support the sense of belonging and workplace activities help to form the professional identity of the learner, even at the initial stage [14]. Furthermore, in order to improve workplace learning in clinical settings, faculty development programs for clinical educators can be arranged focusing on social learning theories, so that the faculty is well oriented and support these learners in this transition phase [15].

As workplace placements are a new addition to the curriculum, this study intends to explore students’ experience of their clinical learning environment and obtain valuable feedback to further improve their training and learning [16]. This study was conducted to see how this experience has been for the students being part of the workplace environment and how it will be useful for them in transitioning to the clerkship phase. This study was conducted using focus group interviews which consists of the collection, analysis, and integration of the findings qualitatively to obtain a deeper understanding of the clinical learning from the students’ perspective. This will support local course management and clinical educators in gaining better understanding of the processes and make changes to improve the learning process for students in the clinical learning environment. As this is a recently introduced course it will help to view its strengths and its potential challenges with relevant suggestions to further improve it. This will enlighten stakeholders all over the world who wish to start this training or are already in the process. The goal is to improve effective patient care through these future practitioners.

## Objective

The research objective is to explore pre-clerkship students’ experiences of learning in the clinical environment in the UAE. It aims to explore students’ experience of various domains of their clinical learning workplace, regarding workplace environment and learning opportunities and extract valuable feedback to further improve their CLE. This study was conducted with the pre-clerkship students before they progress to the clerkship phase of their study. Workplace learning was introduced in their curriculum so that they get an early exposure to the workplace environment which will help them to glide into a smooth transition into their formal clerkship phase.

## Methods

The study setting is Gulf Medical University, UAE which is affiliated to a private teaching hospital and outpatient clinic. The pre-clerkship phase consists of second and third year MBBS where the students will undergo clinical postings daily for four hours during their typical week at the hospital and are rotated in all the major departments, namely medicine, surgery, gynecology, and pediatrics. The students are posted in outpatient clinics across the city where they interact with patients. Workplace learning was recently introduced for second and third year MBBS students which provide opportunities for students to apply and perform the skills like history taking and physical examination learnt in the simulated setting at their university, on real patients under supervision and observe the clinicians interact with the patients in the clinics. All students undergo structured certificate courses in Basic Surgical Skills, Basic Orthopedic Skills and Universal Standard Precautions at the Skills Development Center as part of this course. They are also trained in the American Heart Association accredited Basic Life Support (BLS) Provider course. This course prepares the students to integrate into the hospital settings and perform professionally during the clerkships in years 4 and 5 and during internship.

The main outcomes for the current curriculum of these pre-clerkship students for workplace learning are:

- Demonstrate knowledge of the common clinical conditions encountered in the outpatient setting.
- Apply the basic science knowledge in the organ-system courses to clinical contexts.
- Communicate effectively with patients, family, peers and mentors in the clinical setting.
- Perform clinical skills like history taking, general examination and focused systemic examination.
- Appreciate the importance of maintaining patient medical records.
- Observe management of common clinical conditions encountered in the outpatient setting.
- Reflect upon the experiences during clinical observations and / or learning for self-improvement.

Workplace learning was introduced for the students at our university to facilitate their entry into the clerkship phase. It is important that a thorough benchmark evaluation is done for the clinical learning environment to measure the quality of training from various aspects, and further steps can be planned to make workplace learning a better experience for the learners. Therefore, this study aimed to learn about this phenomenon in depth by using qualitative methods (focus groups). These methods help to study complex phenomena in their natural context. Since this study has a descriptive design and aims to explore the perspective of students about the clinical learning environment that they experience; the theory of phenomenology was applied here. Phenomenology involves data collection from individuals who have lived through the experience of the phenomenon being studied [17]. This aligned with our goals to understand and construct meaning from the lived experiences of students [18].This approach deepened our understanding of the complex phenomena involved in learning, behaviours and communication which occupy centre stage in this critical transition between preclinical to clinical phase [19]. Our goal was to understand the meaning participants attribute to their experiences of preclinical training and this led enhanced understanding and suggestions to improve the status quo. A focus group method was utilized as it provides a robust and interactive discussion between participants that can trigger exploration of contrasting opinions and reflection on common practices. It was considered to conduct group interviews because they are a quick and feasible way to collect data from many people concurrently. Focus groups use group interaction as an important aspect and therefore were considered to be the appropriate data collection method for this research [20, 21]. 

The study was conducted on the students of the pre-clerkship phase (second and third year MBBS). The purposive sampling technique was used so that students from each clinical posting site can be selected to give an overview of the clinical learning environment. Each focus group had eight to ten students [22, 23]. The groups were large enough to allow for varying perspectives and small enough so that everyone could actively participate [24, 25].

### Data collection

The students were informed about the study via email by the department secretary. A mutually convenient time was agreed with the students to collect data so that their learning activities were not affected. The purposive sampling technique was used so that students from each clinical posting site can be selected to give an overview of the clinical learning environment. Thus, it would be representative of the target population [22]. 3–4 students were identified from each clinical group to be included in the focus group discussions. Some students regretted it and finally the first group comprised of ten students and the second group comprised of eight students. The participants were provided with an information sheet which they read and signed on the consent form [25].

The focus group discussions were moderated by me, the author and my co-author was also present with me in these interviews to help me facilitate the discussion. The second co-author was present to observe the discussion. The discussions were tape-recorded & transcribed. Preceding the interview, key trigger questions were devised to facilitate the discussion. I was responsible for putting up the trigger questions and facilitating the discussion, while my co-author helped me record the session, took notes and supported me in summarizing the discussion towards the end of the session, along with debriefing. Each group discussion took between sixty to ninety minutes, depending upon the degree of the discussion [25, 26]. It was ensured that all members had an equal chance of expressing themselves to ensure data reliability. A non-judgmental attitude, having little moderator control was adopted so that the participants could have an honest and interactive dialogue [14]. The findings of the focus group discussion were summarized at the end and the participants were asked to review them. This is known as member checking and helps to strengthen the findings of the discussion [22]. Debriefing was done immediately after the interview with my co-authors, which helped to analyze the data smoothly [27].

The number of focus groups reached adequacy when no new information was collected from the participants, after interviewing two student groups. This is called data saturation as any more information obtained would be redundant [26].

### Data analysis

Krueger’s framework analysis was selected as it guides in a stepwise fashion to analyze qualitative data efficiently for focus group [25].

**Fig. 1:**
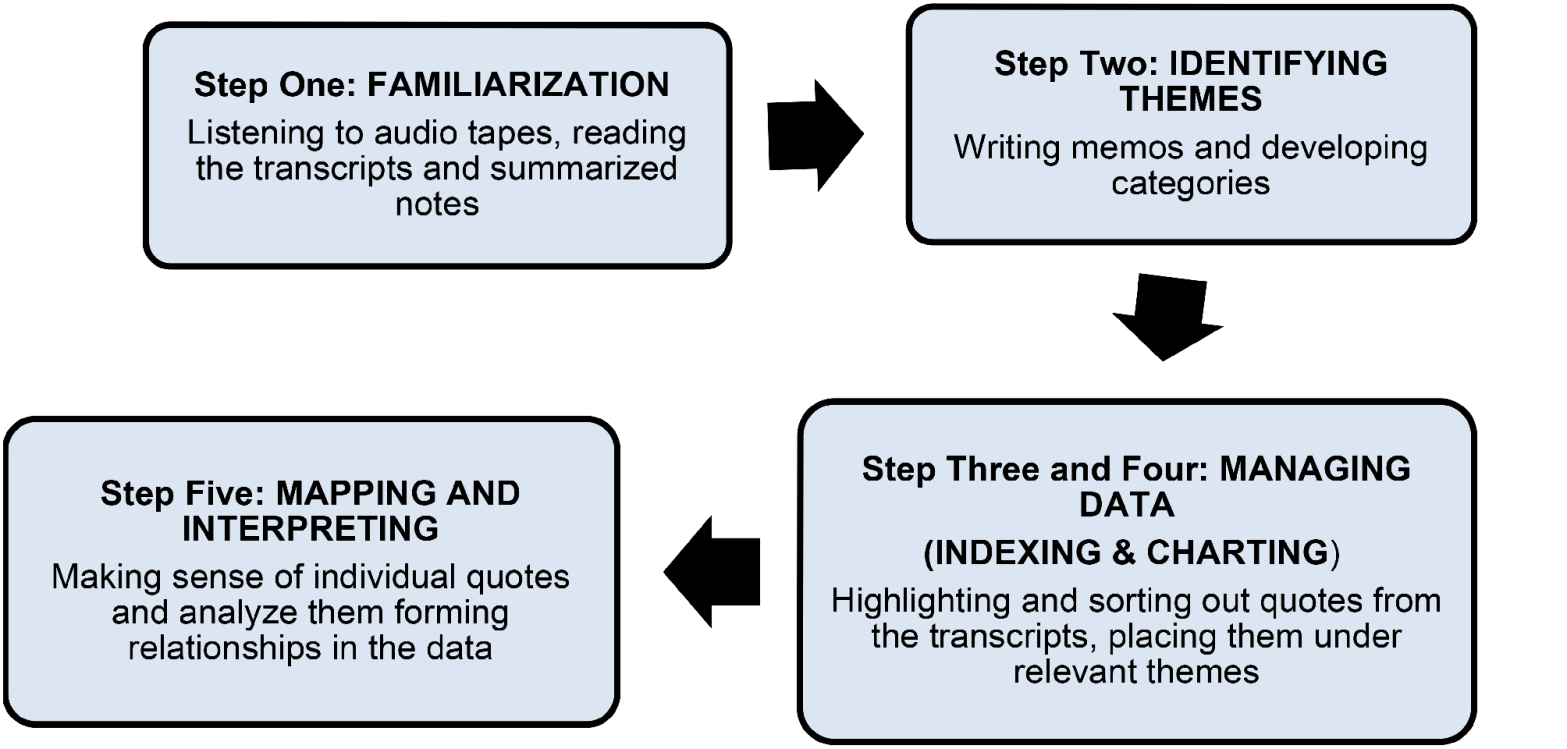
Thematic Analysis (Krueger and Casey, 2009)

Ritchie & Spencer has defined ‘Framework analysis’ as an analytical process involving separate, interconnected steps (Fig. 1). These are familiarization, identifying themes, indexing, charting, mapping, and interpretation [26]. The strength of framework analysis is that it permits themes to develop from research questions and the participants’ responses [22].

Data analysis started during the discussion by combining the narratives with the observation notes, together with my co-authors. We went over the notes, listened to the recordings and ensured that all points were transcribed without missing any points. Familiarization was done by listening to the audio recording, reading the transcripts while referring to the summary of the discussion made post-interview. This helped us to get an overview of the major themes. The codes were identified by highlighting words and phrases of the narrative and categories were made. Indexing and charting, which is called data management collectively was done next and included organizing data, by comparison, highlighting, and placing them under the newly formed themes. This led us to the final steps where the mapping and interpretation which was analytical to establish links in the collected data. Krueger’s criteria to interpret data were followed which guided to look at frequency; specificity; emotions; extensiveness (intensity of comments and big ideas) and the big picture in the data [24]. The coding of the participants is shown in Table 1.

## Results

**Fig. 2:**
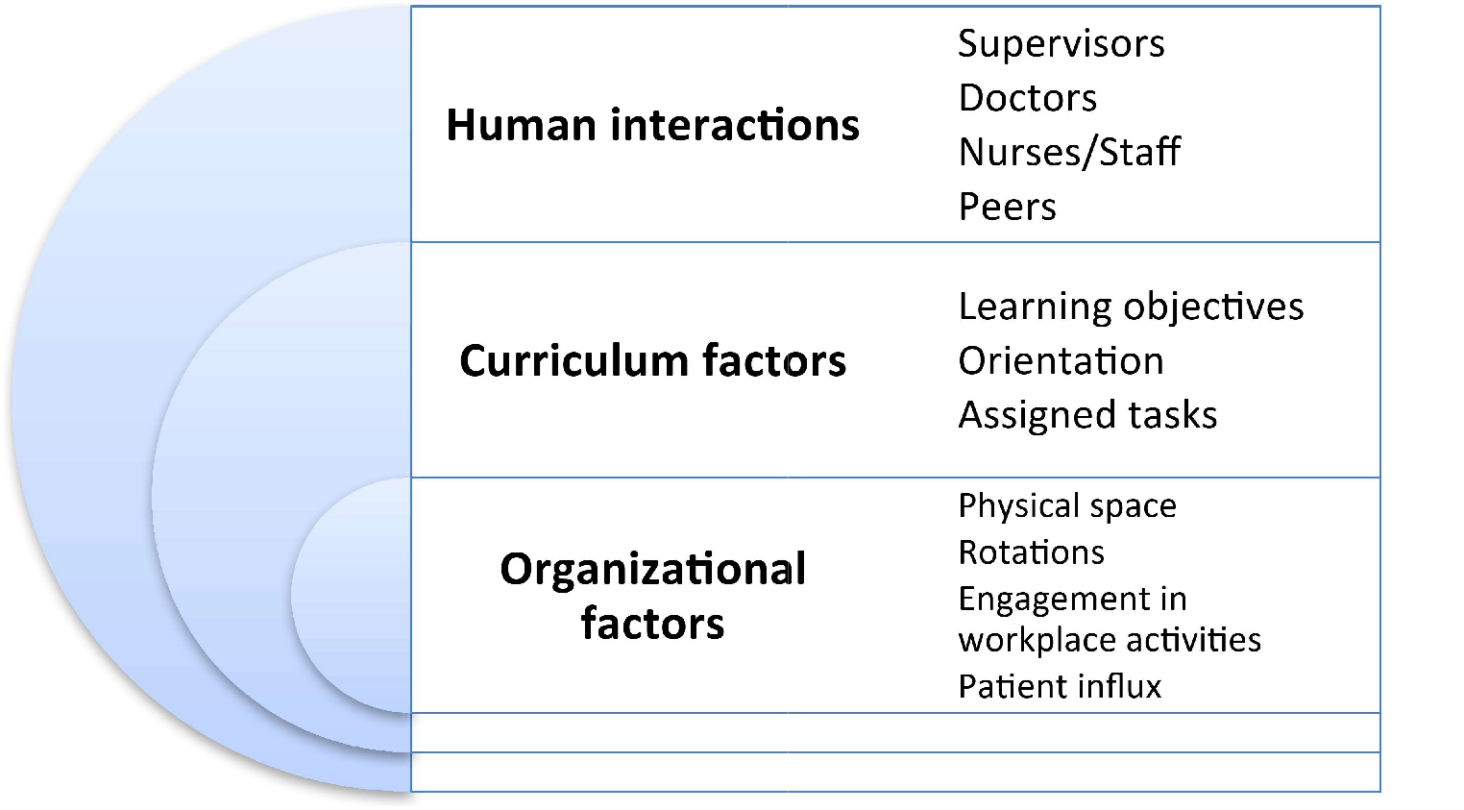
Factors affecting the clinical learning environment

Table 2 illustrates the various themes and categories within those themes that emerged as a result of data analysis. These themes are discussed systematically, supported by actual quotes from the participants. The themes were derived based on factors which influence the clinical learning environment as put forward by Dornan et al., 2007 (Fig. 2) [5]. These factors are aligning well with the themes generated during the focus group interviews. These broad factors are the human factors, curricular factors and organizational factors and are briefly discussed as follows:

### Factors influencing the clinical learning environment

#### Supervision (human factors)

High-quality supervision is an important dimension of workplace instruction, in addition to getting access to patients and promoting independence. High-quality supervision helps students learn independently by interacting with the patients [28]. Sympathetic feedback from the supervisor has a positive influence on the students’ learning and enhanced academic performance [15]. The results that were generated from the focus groups also highlighted the fact that effective supervision tends to have a positive impact on the students and improves the clinical learning environment making it more conducive to learning. This holds especially true for these pre-clerkship students who have been exposed to this new environment and will lead to their motivation and active participation and interaction with the patients. Effective supervisors need to be good role models, be clinically competent and knowledgeable.Some important features of good quality supervision include good interpersonal communication skills, teaching skills, and the ability to offer constructive feedback, which the students have also emphasized upon in the interviews [29].

#### Preparedness of students’ entry (curricular factors)

A clinically oriented introductory period can ease students’ entry into clinical practice, improve their ability to take part in clinical activities, and diminish the anxiety associated with the progress to clerkships [3]. Organizational efforts to improve clerkship transitions include clarifying roles, encouraging socialization and using faculty to offer support to the students in order to dissipate students’ anxieties. The importance of belongingness has been stressed in undergraduate health professions education because it has great influence on the well-being and performance of students [30]. The students in the focus groups have expressed their thoughts that a proper orientation to the students and staff about their postings will help create a sense of inclusion into the clinical workplace and promote their participation further. Belongingness has been understood as being connected or accepted by others through the interaction between learners and people around them in a certain environment [31]. Therefore, it is important to understand the perception of belongingness by the students as it points towards their workplace engagement [32].

#### Participation & interaction (organizational factors)

Workplaces embody socially constituted learning spaces where participatory practices are key pedagogical strategies [6]. The students learn through active participation and interaction with the workplace community in the clinical setting [33]. The motivation of clinical staff towards the educational support of learners, the welcoming attitude of interpersonal relationships in the clinical workplace, the availability of suitable resources are various ways of nurturing student participation [34, 35].This has been further indicated by the students during the focus group sessions that encouragement of participation and interaction with the workspace staff further encourages students to make meaningful contributions and the postings worth attending.

### Theme one: preparedness of student entry (human & curricular factors)

The first theme about how the workplace is prepared to receive the students consists of the orientation of the doctors and staff and the workplace environment which should be conducive to learning.

#### Orientation of the doctors and staff

Students reported that the staff and doctors were expecting the students when they reached there, however the majority of the students in both the focus groups stated that,

*“Doctors were unaware about the year we belong to, had no idea about our learning objectives, and which skills to focus on; some doctors were not expecting us.” (1TY5)*.

*“…nurses and other staff are not oriented; they didn’t expect us and they don’t help us so we feel left out and become uncomfortable.” (2SY6)*.

*“Support staff & nurses…. had a welcoming attitude but they were initially not aware about students coming there and their purpose of visit.”* (*1TY6*).

#### Workplace environment

Students regarded the workplace environment as friendly and highly interactive, where the doctors and staff were easily approachable.

*“Got a good chance to be exposed to this learning environment. Doctors teach us at every opportunity and the environment is good and friendly. They tell us about cases, very interactive.” (1TY3).*

*“Doctors and staff were helpful…everything about this clinic gave me a wonderful opportunity to learn.” (2SY2)*.

### Theme two: learning opportunities (organizational factors)

The second theme which was identified during the interviews was the learning opportunities which included factors like engagement of students in workplace activities, patient influx, rotation schedules and availability of physical space in the workplace.

#### Engagement in workplace activities

The students said that most doctors would allow them to interact with the patient. Students were exposed to real-life situations, and this supported their development of gaining confidence in their approach toward patients.

*“Doctors make sure that whenever there is an interesting case, they will teach us and tell us about the next visit for follow-up.” (2SY3)*.

*“We learned the importance of patient documentation and electronic logs. Common diseases like diabetes, hypertension is there so it gives us a chance to become thorough with those diseases.” (2SY8)*.

*“Nurses could help or assist in the initial assessment of the patient so that students feel part of the team.” (1TY8)*.

#### Physical space

Students reported in the focus groups that the clinics are small and there is hardly space for 2–3 students to sit or even stand sometimes. The students said that due to lack of space they keep standing for almost four hours, which is uncomfortable and they start losing interest.

*“There were no chairs; we were standing for a long time about four hours.” (1TY1)*.

*“There is no proper place for us to sit and discuss our findings and case with doctors.” (2SY8)*

#### Patient influx

The patient influx for most of the morning postings was very low. Students who were posted in the evening had more patient influx than in the morning. The students also pointed out that the general practitioners don’t have a variety of cases,

*“Some clinics had a good influx of cases and have doctors who are willing to teach…some clinics will have very few doctors, so it’s not a fair share for all students as they are rotating in the same site for two years.” (1TY6)*.

*“The general practitioners don’t have time to discuss with us as they have many cases; there is no variety and cases are repeated a lot.” (2SY6)*.

*“Limited cases at few places…the patient flow is very low and repetitive cases are present. Sometimes there are no patients. “(1TY3)*.

#### Language barrier

This is a common challenge reported by many students. The doctors and nurses don’t have time to translate or summarize the case for students as all of them are not Arabic speaking students. The students are not able to follow what is happening and find this very frustrating.

*“Some of the doctors would summarize /translate their conversation with the patient for us, as quite often patients and doctors speak a language different than English. Some will not do so, due to lack of time*.” *(1TY4).*

*“If the doctor and patient speak the same language, students cannot understand what’s going on;… it gets worse if the nurse is also speaking in the same language.” (2SY7)*

#### Rotation schedules

There should be proper schedules to which doctor the student should be reporting to at each clinic.

*“There were no proper schedules for where we need to go, we could choose our own doctor. There were no assigned doctors for us.” (1TY4)*.

*“Especially in evenings, there are a lot of patients, in the mornings patients are few, and most of the posting time we are free.” (1TY6)*.

### Theme three: quality of supervision (human factors)

The students expressed their views that the supervisors are good and approachable. General feedback is given; however, there is no structured feedback session and only provided upon prompting.

*“Doctors are quite busy in their schedules so no time for proper feedback. If we don’t ask about it, we don’t get it.” (1TY10)*.

*“No structured or written feedback.” (2SY8)*

#### Possible solutions to the identified challenges

Discussed below are possible solutions to the challenges commonly experienced by students which emerged from the focus group discussions.

### Theme one: preparedness of student entry

#### Proper orientation of the staff

There can be proper orientation programs for the doctors and healthcare staff regarding the students’ postings and their learning outcomes. The learning objectives for students could be aligned with those of the university so that the learning is contextualized.

*“We need doctors to guide us what to do in clinics and how to make our postings more productive. For that they need to be properly oriented and trained.” (2SY1)*.

*“…………. the doctors should be well oriented, they should have more workshops or training in order to be well-prepared to receive the students.” (1TY4)*.

*“Doctors should be well aware of our training level and learning objectives.” (1TY1)*.

### Theme two: learning opportunities

#### Language barrier:

Doctors or nurses could summarize the case for the students so that they are in the loop during doctor-patient interaction.

*“They should also do a quick translation after the patient has left so that we have the opportunity to learn something.” (2SY7)*

#### Patient influx and rotation schedules

The students suggested that instead of morning, evening rotations could be scheduled as more patients are there in the evening.

*“Hospital postings would be a good idea in addition to clinics; maybe we will get more opportunities. Other universities have postings in hospitals.” (2SY5)*.

*“We should get chances to switch from one clinic or other or we should be rotated periodically to get fair chance to see all kinds of patients and interacting and benefitting with so many clinicians.” (2SY6)*.

*“Evening timings are preferred because of more patients. Till 12 noon there are no patients.” (2SY7)*

#### Physical Space

There should be separate discussion rooms available for students where they can discuss the cases with their peers and doctors.

*“Each clinic should have separate discussion rooms.” (2SY3)*.

*“Proper discussion rooms should be there in each clinic as many students are not allowed in the doctor?s room because of infection control.” (1TY3)*.

*“Evening timings are preferred because of more patients. Till 12 noon there are no patients.” (2SY7)*

### Theme three: supervision

There should be proper and structured feedback sessions scheduled in which the student and supervisor can discuss the progress of the student.

*“Feedback should be formally given and scheduled so that we learn about our gaps.” (2SY4)*.

*“There is hardly any feedback so, we decided to set our own goals and direction, helped us to become self-directed learners.” (1TY2)*

## Discussion

The focus groups explored thoroughly the students’ perceptions regarding their learning opportunities, level of participation and supervision. The key findings which were revealed during the focus groups highlight the importance of the preparedness of the students’ entry into the workplace which included the orientation of the staff about the expected outcomes of the students. The findings of this study also emphasize on the fact that efforts can be made to improve the quality of supervision and increase the number of learning opportunities for the students, to shape the clinical learning environment more educationally conducive for them [2].

The students will also benefit if a proper instructional design strategy can be incorporated in this part of the curriculum i.e. pre-clerkship postings. This can be done through one of the common instructional design models, known as ADDIE. It stands for Analysis, Design, Development, Instruction and Evaluation [36] This can be utilized as follows:

## Addie Model

1. Analysis: As discussed in the introduction, the need for introducing pre-clerkship training before the actual clerkship for undergraduate medical students was important in order to alleviate the anxiety and stress associated with this major transition of students’ lives.
2. Design: The clinical postings were designed in such a way that the students would be spending four hours at their respective posting in a typical week. They will be rotated in all major departments.
3. Development: The students will be spending their time at the postings by being attached to their supervisors who will be assigning them tasks for each day. The students will be interacting with the patients, the staff and the doctors and all these organizational factors will help them feel a part of the workplace community and create a favorable learning environment.
4. Implementation: For effective implementation, the staff and the doctors will need to be oriented to the students postings and their objectives. Their positive and welcoming attitude will encourage students to participate in the workplace activities. The administrator needs to make sure that students are attending their postings regularly and following the given timings.
5. Evaluate: This can be evaluated at the end of the academic year using the Kirkpatrick model. The effectiveness can be evaluated during the implementation stage where the students’ reaction and learning can be seen and then summative at the end of the academic year to look at their behavior and the outcome of this pre-clerkship course [37].

These findings of the focus group interviews are discussed below thematically in detail along with the potential solutions to the challenges faced by the participants to improve the CLE (Fig. 2).

### Preparedness of student entry (human & curricular factors)

The students reported that their clinical learning environment was student-friendly and the doctors and staff were easily approachable. The students described that though they had a welcoming attitude, most of the doctors and staff were not aware of the students’ postings and students’ training level and learning objectives. This highlights the significance of a clinically oriented introductory period which would facilitate students’ entry into the clinical environment and motivate them to participate in workplace activities [1, 3]. Atherley et al. advocates that it is necessary to have formal orientations and staff training so that they would be better equipped to receive the new learners [38]. Students also expressed their feeling of lack of being part of the team. A sense of belongingness to the workplace and identity formation of students as individual learners can be created by facilitating the interpersonal relationships at the workplace, having supervisors with a welcoming attitude, healthcare staff being supportive and having a good mentorship for which their seniors can play a definitive role [35, 39].

#### Potential solutions

Students suggested that a formal orientation should be arranged by the clerkship directors for the doctors and healthcare staff so that they are aware of the students’ training purposes and learning outcomes [2]. Workshops can be conducted for the doctors and nursing staff, where a welcome introduction to the department is given, students’ roles and responsibilities and supervisor expectations are clarified [38]. In this way, socialization will also be promoted and students’ apprehension due to transition will be considerably reduced.

### Learning opportunities (organizational factors)

During focus group discussions, many students reported that most often doctors allow them to interact with the patient and involve them in follow-up and documentation. They also revealed the nursing staff as cooperative that supported students’ learning by involving them in various patient care tasks. At other sites, students experienced less encouragement to participate and could not get adequate support from the staff. Participation relies heavily on the interactions between the staff and learners as it strongly influences undergraduate medical students’ success [40]. Participation can be improved by the motivation of clinical staff towards the students’ educational needs, providing adequate resources, and creating opportunities to support their learning [34, 35]. The link between favorable learning climates and participation in the workplace has been strongly supported by studies of undergraduate medical students in workplaces [41].

Students suggested a discussion room to be available for students where they could discuss the cases with their peers and doctors. Many studies have emphasized the importance of the physical condition of the clinics and the availability of space as important constituents of favorable clinical learning environment [35, 40, 42].

It was pointed out by most students that the patient influx is more in the evening than in the morning. Students also pointed out that they should be posted in hospitals in addition to the clinics, to have a wider exposure of patients. This aspect of the clinical learning environment is well-established in literature that access to appropriate patients is an important dimension of workplace instructional quality [28, 41].

Another point raised by the students is that the majority of the students faced problems due to the language barrier. In the UAE, the local language is Arabic so many patients speak the same language. Although the official language for communication is English, but in this multicultural country where most of the patients are from the Middle East, they mostly communicate in Arabic. If the doctor, nurse, or the patients are communicating in the same language, unknown to the students, it becomes difficult for the students to understand the conversation.

#### Potential solutions

The learning opportunities can be increased for the students by providing access to more patients, encouragement to participate, strengthening interaction with the workplace team, provision of physical space, and overcoming the language barrier for certain interactions [6, 38]. The nurses could involve them in the initial assessment of the patient, which helps to develop an identity and the students participate as a team member in imparting patient care [41].

Regarding the language barrier, Sreekanth claims that the Google language tool (GLT) can improve the communication between patient and doctor [43]. Another way is to make student group combinations in such a way that they can help each other in understanding the language in clinical placements, which can be viewed as formal peer-to-peer learning. This denotes that formal student support systems play a significant role in dealing with many of the challenges that students face in their learning environment [44]. It can be done by providing mentorship to students involving the faculty and seniors [45]. This would help to alleviate the students’ stress, promote socialization, and further optimize the learning environment [38].

### Quality of supervision (human factors)

Students reported that there are very few structured feedback sessions which help the students to improve their learning. It made the students feel doubtful about themselves if they were able to achieve their outcomes or not [46]. This highlights the significance of proper orientation of the doctors and its strong correlation with the quality of supervision and feedback. Immediate and constructive feedback should be provided before, during, and after the rotation to build a supportive learning environment. Literature has almost always emphasized that quality of supervision has a significant impact on medical students’ learning in the clinical workplace [29, 42].

#### Potential solutions

The students reported that supervision be improved by providing regularly scheduled structured sessions and provide constructive feedback to the students to improve their learning. The supervisors should also be well aware of the local training bodies’ requirements and guide students accordingly [30].

### Recommendations for practice

**Fig. 3:**
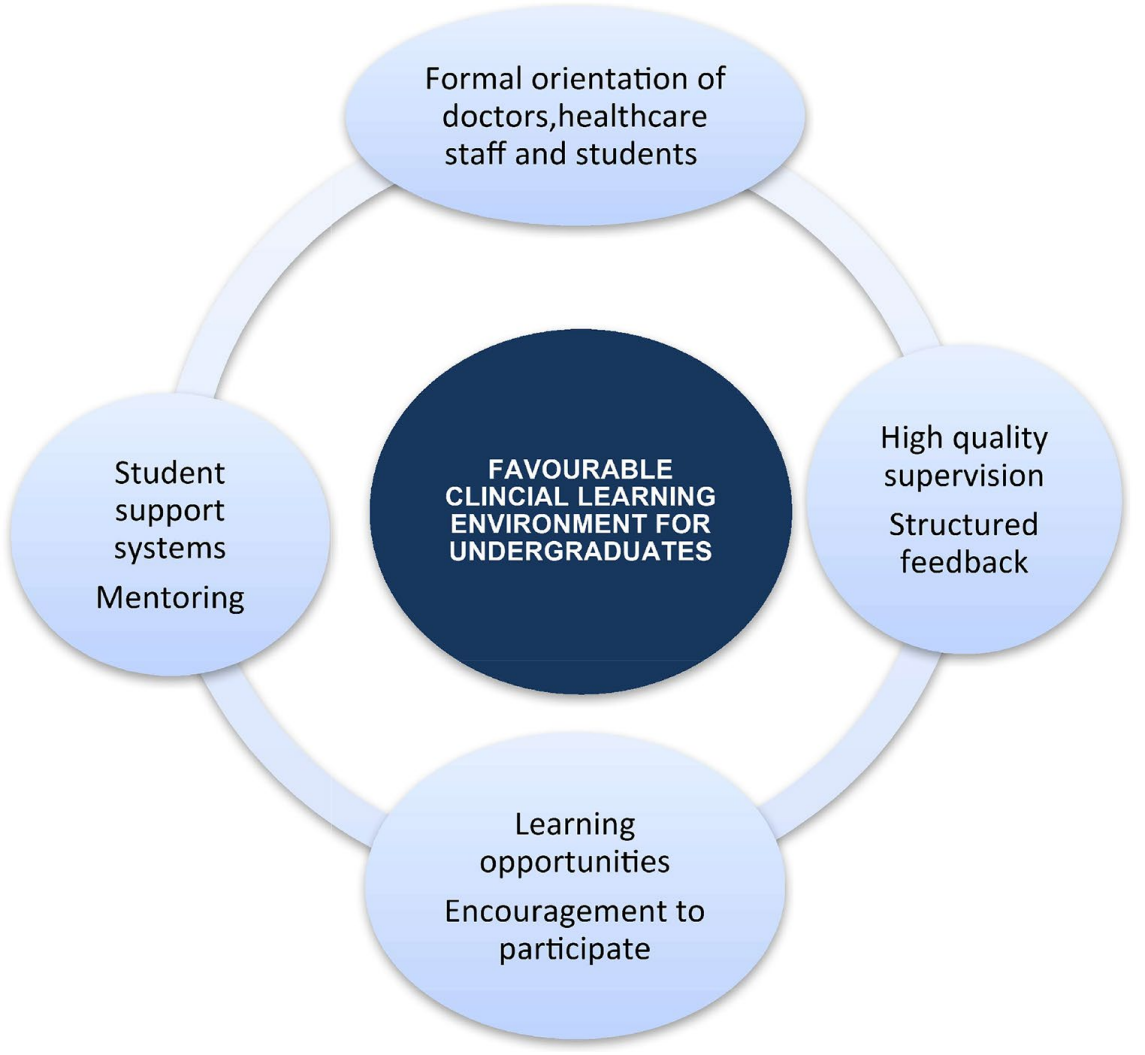
Recommendations for practice

The findings of this study could be utilized by curriculum leaders and stakeholders in UAE and the region beyond because it has unfolded many important factors and suggestions about the CLE. Based on the results of this study, the learning environment for students at the clinical workplace can be optimized in the following ways (Fig. 3).

#### Formal orientation programs

Students suggested that a formal orientation should be arranged by the clerkship directors for the doctors and healthcare staff so that they are aware of the students’ training purposes and learning outcomes [47]. This would also help to create multiple learning opportunities for the new learners as they gradually become an integral part of the clinical workplace [38]. A way to ensure this is that workshops and training can be conducted for the doctors and nursing staff, a welcome introduction to the department, where students’ roles and responsibilities and supervisor’s expectations are clarified. In this way, socialization will also be promoted and students’apprehension due to transition will be considerably reduced.

#### Increased Learning Opportunities

The postings should include hospitals and students should be rotated at all sites equally. Evening postings are more appropriate due to the larger patient influx at that time; as patients provide the most important learning prospects for the students [38]. The importance of discussion rooms and physical space is also stressed by the students. Participation and learning processes are mutually dependent and learning through involvement in daily practices shapes the learning process and desired outcomes [6]. Therefore, encouraging students to participate in different aspects of patient care can make them feel part of the team.

#### Student support systems

Another important recommendation to encourage socialization is by providing formal student support [38]. This can be done by developing strategies for providing mentorship to students and forming student support systems involving the faculty and seniors [45]. This role can be appointed to the existing training residents who can schedule meetings with the students at regular intervals. This would help to alleviate the students’ stress, promote socialization, and further optimize the learning environment.

#### High-Quality supervision

The students also stressed the importance of high-quality supervision as it shapes the learning process of the students and helps to identify their gaps. To implement it, supervision should include structured and constructive feedback to the students to improve their learning and there should be a self-reflective practice from both supervisors and students [38].

### Implications of the study

This study holds a reasonable prospect that the populations in which the research is carried out stand to benefit from the results of the research, and the future students. Other stakeholders may be included such as the clerkship directors and clinical educators. They are also interacting with these students and can give an insight into the common problems encountered by the students and their probable solutions [11]. These students’ perceptions may vary over a period of time once they proceed to the clerkship phase. Data from the same students may be collected in the clerkship stage so they are better able to identify the challenges of this course. This would make the current course better, while at the same time laying strong foundations for the clerkship phase training.

## Conclusion

The perception of students was generally satisfactory towards their workplace environment. The students felt welcomed, were encouraged to participate in workplace tasks and found the interaction with their supervisors and healthcare staff appropriate. However certain factors like improved learning opportunities, proper physical space, low patient influx at some sites and difficulty communicating with patients in their language were explored in depth during focus group discussions. These discussions brought into light the potential measures that could be taken to further strengthen the learning environment for the learners. The findings of this study could be utilized by curriculum leaders and stakeholders in this context and beyond the region because it has unfolded many important factors and suggestions about the CLE. This study will become one of the types of evidence in order to implement this training course for medical undergraduates and also get ideas to improve certain aspects where this course is already implemented.

**Table 1:** Coding of participants during focus group interview

Focus Group One 3rd year	Focus Group Two 2nd year
1TY1	2SY1
1TY2	2SY2
1TY3	2SY3
1TY4	2SY4
1TY5	2SY5
1TY6	2SY6
1TY7	2SY7
1TY8	2SY8
1TY9	
1TY10	
10 students	8 students

**Table 2:** Themes and categories from qualitative data analysis

Themes	Subthemes	Codes	Potential solutions
Preparedness of student entry (Human & Curricular factors)	Orientation of the doctors Orientation of the staff	Lack of orientation of doctors and support staff They should be sure of the extent to which they could allow the student to deal with the patient	Proper orientation of the doctors, nurses and support staff Workshops and training for doctors Alignment of the learning objectives with that of university One point of contact for each outpatient department
	Workplace environment	Welcoming attitude but unaware of the purpose of students’ postings Cooperative Interactive and friendly environment Doctors are approachable	
Learning opportunities (Organizational factors)	Engagement in workplace activities	Doctors try to engage us in interesting cases Willing to teach Help to identify gaps Documentation,electronic logs	
	Physical space	Well organized setup Clinics are small; get easily crowded, a standing for hours No place to sit and discuss the case with doctors	Separate discussion rooms can be made available.
	Patient influx	GP has, repetitive cases Specialists have different cases but less time for teaching	
	Language barrier	If the doctor and patient have the same language, difficult for students to understand No time for translate	Doctors or nurse could summarize the case for us
	Rotation schedules (days & timings)	Morning postings, very few patients, till noon Rotation at only one site for the whole year No specific clinic schedules for students	Postings can be scheduled in the evening as more patients Students should be rotated across all sites Hospital postings could be added Properly assigned clinic
	Access to patient records	Difficulty in accessing medical records	Special access to students can be given
Quality of supervision (Human factors)	Supervisory relationship	Try to involve us in case discussion Friendly doctors Good rapport with them	Can give us more hands-on so that students feel part of the team
	Feedback by doctors/supervisors	Informal Upon prompting	Structured feedback with one to one discussion to identify gaps and improve performance

## Data Availability

The datasets used and analyzed during the current study are available from the corresponding author on request. It includes the transcript of the focus group discussions.

## References

[1] Teunissen PW, Westerman M (2011). Opportunity or threat: the ambiguity of the consequences of transitions in medical education. Med Educ.

[2] Gallagher P, Carr L, Wang SH, Fudakowski Z (2012). Simple truths from medical students: perspectives on the quality of clinical learning environments. Med Teach.

[3] Poncelet A, O’Brien B (2008). Preparing medical students for clerkships: a descriptive analysis of transition courses. Acad Med.

[4] Evans K, Guile D, Harris J, Malloch M, Cairns L, Evans K, O’Connor B (2011). Rethinking work-based learning: for education professionals and professionals who educate. The Sage Handbook of Workplace Learning.

[5] Dornan T, Boshuizen H, King N, Scherpbier A (2007). Experience-based learning: a model linking the processes and outcomes of medical students’ workplace learning. Med Educ.

[6] Billett S (2004). Workplace participatory practices: conceptualising workplaces as learning environments. J Workplace Learn.

[7] Morris C, Behrens M, Kieran, Walsh (2013). Work based learning. Oxford textbook of medical education.

[8] Yardley S, Teunissen PW, Dornan T (2012). Experiential learning: transforming theory into practice. Med Teach.

[9] Vygotsky LS, Cole M. Mind in society: development of higher psychological processes. Harvard University Press; 1978.

[10] Scott SE, Palincsar AS. Sociocultural theory. I: M. Anderman & LH Anderman (Red.). Psychology in Classroom Learning: An Encyclopedia. 2009.

[11] O’Brien B, Cooke M, Irby DM (2007). Perceptions and attributions of third-year student struggles in clerkships: do students and clerkship directors agree?. Acad Med.

[12] Lave J, Wenger E. Situated learning: legitimate peripheral participation. Cambridge University Press; 1991.

[13] Fuller A, Unwin L. 2006. Expansive and restrictive learning environments.

[14] Wenger E (1998). Communities of practice: learning as a social system. Syst Think.

[15] Morris C, Blaney D, swanwick T (2010). Work-based learning. Understanding Medical Education: evidence, theory and practice.

[16] Soemantri D, Herrera C, Riquelme A (2010). Measuring the educational environment in health professions studies: a systematic review. Med Teach.

[17] Ng SL, Baker L, Cristancho S, Kennedy TJ, Lingard L. Qualitative research in medical education: methodologies and methods. Understanding medical education: Evidence, theory, and practice. 2018:427– 41.

[18] Teherani A, Martimianakis T, Stenfors-Hayes T, Wadhwa A, Varpio L (2015). Choosing a qualitative research approach. J Graduate Med Educ.

[19] Neubauer BE, Witkop CT, Varpio L (2019). How phenomenology can help us learn from the experiences of others. Perspect Med Educ.

[20] Kitzinger J (1995). Qualitative research: introducing focus groups. BMJ.

[21] Brown JB, Crabtree BF, Miller WL (1999). The use of focus groups in clinical research. Doing qualitative research.

[22] Tavakol M, Sandars J (2014). Quantitative and qualitative methods in medical edu-cation research: AMEE Guide 90: part II. Med Teach.

[23] Starks H, Brown Trinidad S (2007). Choose your method: a comparison of phenom-enology, discourse analysis, and grounded theory. Qual Health Res.

[24] Krueger RA (2009). Focus groups: a practical guide for applied research.

[25] Rabiee F (2004). Focus-group interview and data analysis. Proc Nutr Soc.

[26] Stalmeijer RE, McNaughton N, Van Mook WN (2014). Using focus groups in medical education research: AMEE Guide 91. Med Teach.

[27] Doody O, Slevin E, Taggart L (2013). Focus group interviews part 3: analysis. Br J Nurs.

[28] Van der Zwet J, Hanssen VG, Zwietering PJ, Muijtjens AM, Van der Vleuten CP, Metsemakers JF, Scherpbier AJ (2010). Workplace learning in general practice: supervision, patient mix and independence emerge from the black box once again. Med Teach.

[29] Kilminster S, Cottrell D, Grant J, Jolly B (2007). AMEE Guide 27: effective educational and clinical supervision. Med Teach.

[30] Reay D, Crozier G, Clayton J (2010). Fitting in’or ‘standing out’: Working-class students in UK higher education. Br Edu Res J.

[31] Vivekananda-Schmidt P, Sandars J (2018). Belongingness and its implications for undergraduate health professions education: a scoping review. Educ Prim Care.

[32] Trujillo G, Tanner KD (2014). Considering the role of affect in learning: monitoring students’ self-efficacy, sense of belonging, and science identity. CBE—Life Sci Educ.

[33] Öhman E, Alinaghizadeh H, Kaila P, Hult H, Nilsson GH, Salminen H (2016). Adaptation and validation of the instrument clinical learning environment and supervision for medical students in primary health care. BMC Med Educ.

[34] Stark P (2003). Teaching and learning in the clinical setting: a qualitative study of the perceptions of students and teachers. Med Educ.

[35] Papp I, Markkanen M, von Bonsdorff M (2003). Clinical environment as a learning environment: student nurses’ perceptions concerning clinical learning experiences. Nurse Educ Today.

[36] Allen WC (2006). Overview and evolution of the ADDIE training system. Adv Developing Hum Resour.

[37] Kirkpatrick J. 2015. An introduction to the new world Kirkpatrick model. Kirkpatrick Partners, p.2019.

[38] Atherley AE, Hambleton IR, Unwin N, George C, Lashley PM, Taylor CG (2016). Ex-ploring the transition of undergraduate medical students into a clinical clerkship using organizational socialization theory. Perspect Med Educ.

[39] Levett-Jones T, Lathlean J, Higgins I, McMillan M (2009). Staff–student relationships and their impact on nursing students’ belongingness and learning. J Adv Nurs.

[40] Boor K, Scheele F, Van Der Vleuten CP, Teunissen PW, Den Breejen EM, Scherpbier AJ (2008). How undergraduate clinical learning climates differ: a multi-method case study. Med Educ.

[41] Dornan T, Muijtjens A, Graham J, Scherpbier A, Boshuizen H (2012). Manchester Clinical Placement Index (MCPI). Conditions for medical students’ learning in hospital and community placements. Adv Health Sci Educ.

[42] Dolmans DH, Wolfhagen IH, Essed GG, Scherpbier AJ, van der Vleuten CP (2002). The impacts of supervision, patient mix, and numbers of students on the ef-fectiveness of clinical rotations. Acad Med.

[43] Sreekanth G (2010). The use of Google language tools as an interpretation aid in cross-cultural doctor–patient interaction: a pilot study. Inf Prim Care.

[44] O’Reilly SL, Milner J (2015). Supporting culturally and linguistically diverse students during clinical placement: strategies from both sides of the table. BMC Med Educ.

[45] Bauer TN, Erdogan B, Zedeck S (2011). Organizational socialization: the effective onboarding of new employees. Maintaining, expanding, and contracting the organization: APA handbooks in psychology.

[46] Prince KJ, Boshuizen HP, van der Vleuten CP, Scherpbier AJ. Students’ opinions about their preparation for clinical practice. Med Educ. 2005; 39(7):704– 12. 10.1111/j.1365-2929.2005.02207.x. PMID: 15960791. 10.1111/j.1365-2929.2005.02207.x 15960791

[47] Heidenreich C, Lye P, Simpson D, Lourich M. The search for effective and efficient ambulatory teaching methods through the literature. Pediatrics. 2000 14;105(Supplement_2):231-7. 10617728

